# The Evolution of Transmissible Prions: The Role of Deformed Templating

**DOI:** 10.1371/journal.ppat.1003759

**Published:** 2013-12-05

**Authors:** Natallia Makarava, Ilia V. Baskakov

**Affiliations:** Center for Biomedical Engineering and Technology and Department of Anatomy and Neurobiology, University of Maryland School of Medicine, Baltimore, Maryland, United States of America; Washington University School of Medicine, United States of America

## Prion Strain Mutation and Evolutionary Concepts

The last several years have marked a noticeable shift in our perception of the prion replication mechanism. According to the “cloud” hypothesis, pools of infectious isoform of prion protein (PrP^Sc^) within individual strains or isolates are intrinsically heterogeneous; the heterogeneity presumably arises due to spontaneous variation in PrP^Sc^ structure [1], [2]. Upon changes in the replication environment, minor variants that fit best to replicate in the new environment receive selective advantages. Consistent with this view, a growing number of studies have highlighted the fact that prion strains exhibit high levels of conformational plasticity and are subject to transformation when exposed to new replication environments. Drug-resistant prions were found to emerge in cultured cells following treatment with prion inhibitors swainsonine or quinacrine [2], [3]. Studies by Weissmann's group showed that cloned prion strains accumulate PrP^Sc^ variants quite quickly, presumably due to ongoing processes of spontaneous “mutations” of PrP^Sc^ structure [2].

**Figure 1:**
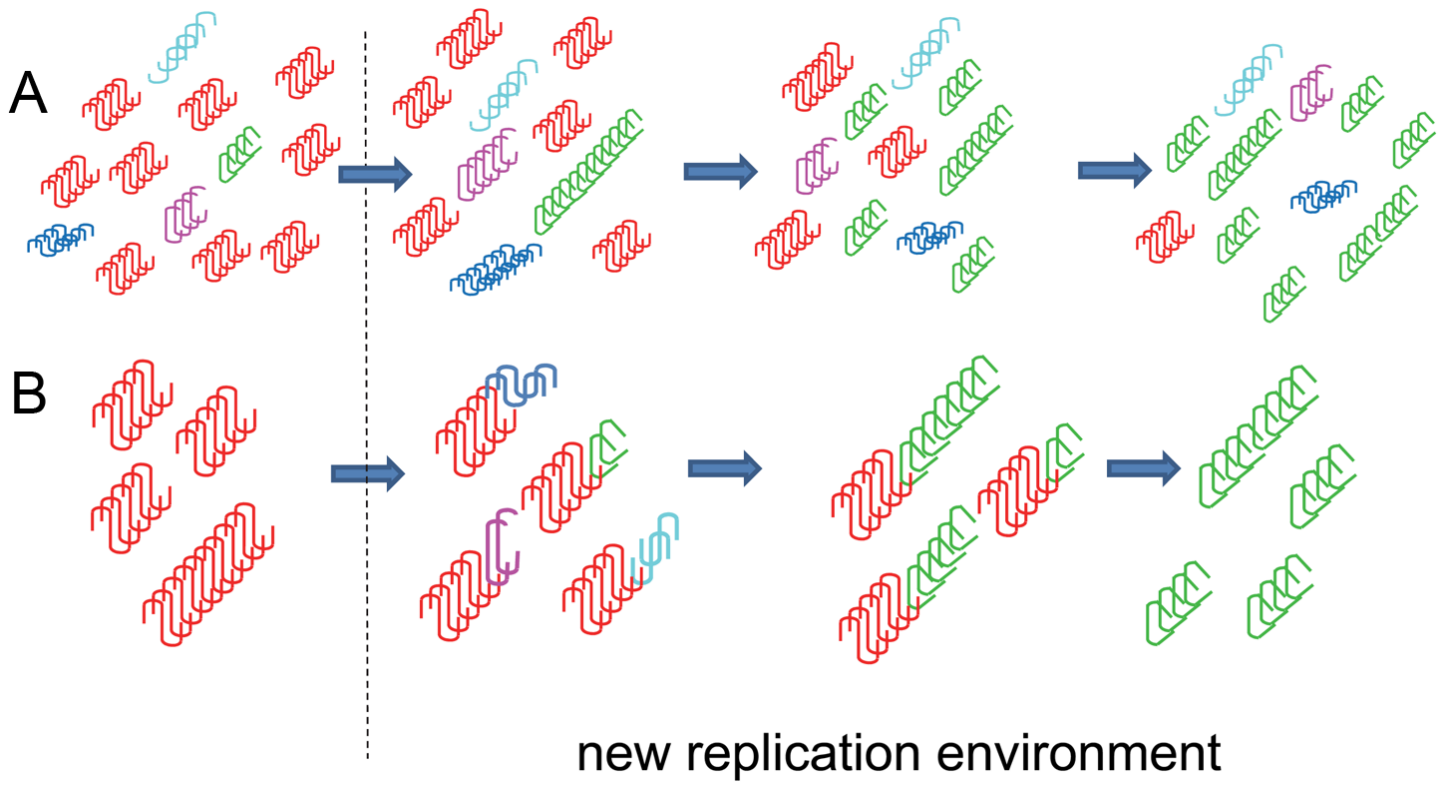
Schematic diagrams illustrating two alternative hypotheses on origin of prion mutations. (**A**) The “cloud” hypothesis proposes that prion isolates are intrinsically heterogeneous and consists of major (red) and minor (various colors) PrP^Sc^ variants. Changes in the replication environment might provide selective advantages for replication of a minor variant leading to transformation of the PrP^Sc^ population. (**B**) The deformed templating model postulates that diverse structural variants are generated as a result of changes in replication environment via numerous PrP^Sc^-dependent trial-and-error seeding events. A newly generated variant that fits better than parent PrP^Sc^ to the altered environment replaces the original PrP^Sc^ variant.

What are the origins of strain mutation and how do prions mutate? According to the “cloud” hypothesis, changes in replication environment might give selective advantage to minor PrP^Sc^ variants that are already present in the PrP^Sc^ pool [1]. However, the origin of minor variants is not clearly specified (Figure 1A).

## Role of Deformed Templating in Prion Mutation and Evolution

An alternative to the “cloud” hypothesis is a deformed templating model that postulates that changes in replication environment play an active role in generating new PrP^Sc^ variants, in addition to its role in imposing a selective pressure (Figure 1B). Even if a PrP^Sc^ template does not fit a new environment, it can still seed new PrP^Sc^ variants via deformed templating. While the majority of the newly generated variants might not be effective in replicating, a variant that fits well to the new environment will eventually emerge through multiple trial-and-error seeding events. Therefore, the change in the replication environment boosts conformational diversity of the PrP^Sc^ pool and selects the variant that is the best fit for that environment.

The two models are not mutually exclusive, and both are likely to be involved in prion evolution. While deformed templating does not argue against structural heterogeneity of a PrP^Sc^ population of natural or synthetic origin, it helps to explain observations that would be difficult to understand solely based on the “cloud” hypothesis, as discussed in the next section. The fundamental difference between the two models is in the origin of altered PrP^Sc^ states. In contrast to the “cloud” hypothesis, deformed templating proposes that changes in replication environment play an active role in expanding the pool of available PrP^Sc^ variants. In addition, deformed templating proposes that new variants emerge in a template-dependent manner, although they do not faithfully reproduce a parent state. We do not know whether deformed templating occurs only when the environment is unfavorable for faithful propagation or whether it can also occur in the absence of changes of environment, but at a lower rate.

Deformed templating postulates that a state with one cross-β folding pattern can seed an alternative self-replicating state with a different folding pattern [4]. Such seeding is possible if the parent and daughter states share common structural motifs that link the hybrid structure. Atomic force fluorescence microscopy imaging provided a direct demonstration that folding patterns can be switched within individual fibrils [5]. Because common structural motifs are shared between daughter and parent states, deformed templating predicts structural continuity or “memory” in the evolution of self-replicating states.

It is difficult to prove experimentally whether new PrP^Sc^ variants appear upon changing replication environment via selective amplification of preexisting minor variants or emerge *de novo* via deformed templating [6], [7]. Nevertheless, recent studies provided experimental evidence that changes in replication environment generate new PrP^Sc^ states. Adaptation of hamster strains 263K or Hyper to RNA-depleted brain homogenates and then readaptation to brain homogenates containing RNA in protein misfolding cyclic amplification (PMCA) was shown to result in a stable change in PrP^Sc^ properties including PK resistance, conformational stability, and amplification rate [8]. Remarkably, upon reversible changes in RNA content, the amplification rate of the newly emerged PrP^Sc^ variants (referred to as 263K^R+^ or Hyper^R+^) was 10^4^-fold higher than that of brain-derived 263K. Further experiments revealed that 263K^R+^ was absent in 263K brain material and emerged *de novo* as a result of reversible changes in replication environment [8].

## Deformed Templating and Evolution of Synthetic Prions

The studies on synthetic prions provided intimate insight into the role of deformed templating in the evolution of transmissible protein states. In the last decade, a number of synthetic strains were generated in animals by inoculating amyloid fibrils produced *in vitro* using recombinant prion protein (rPrP) [9]–[12]. While it is becoming increasingly evident that PrP folding patterns within rPrP fibrils and PrP^Sc^ are fundamentally different [13], the question of greater interest is how noninfectious amyloid fibrils gave rise to PrP^Sc^ and transmissible disease. An exhaustive search for miniscule amounts of PrP^Sc^ in the preparations of rPrP fibrils using PMCA that detects single PrP^Sc^ particles yielded negative results [14], [15]. This argues against the hypothesis that transmissible synthetic strains emerged in animals via selective amplification of minor, PrP^Sc^-like conformations that might have been present in rPrP fibril preparations. An alternative hypothesis postulates that PrP^Sc^ evolved from rPrP fibrils via a series of deformed templating events [4], [15]. Indeed, the first product of PrP^C^ misfolding triggered by rPrP fibrils in hamsters was a self-replicating PrP state (referred to as atypical PrPres) characterized by an abnormally short, C-terminal PK-resistant core similar to that of rPrP fibrils [14], [15]. Unlike hamster PrP^Sc^, atypical PrPres preferred monoglycosylated PrP^C^ as a substrate and its amplification was RNA-independent. Over the course of several serial passages, atypical PrPres gave rise to PrP^Sc^.

According to deformed templating, the parent template and the altered daughter state share common structural motifs. Consistent with this prediction, the study on synthetic strains produced by rPrP fibrils with a range of conformational stabilities revealed a strong correlation between stability of rPrP fibrils and end-product PrP^Sc^ [10]. This correlation would be difficult to explain if one assumes that PrP^Sc^ emerged from a minor subpopulation.

## Strain Adaptation and Deformed Templating

Cross-species prion transmission often causes diseases with a diminished frequency and is accompanied by a prolonged silent stage, a phenomenon known as species barrier. When followed by serial passaging, the incubation time to disease decreases and strain properties change, reflecting adaptation of prions to a new environment. The “cloud” hypothesis attributes prion adaptation to selection of minor PrP^Sc^ variants that replicate better than others in a new host [1]. Studies on synthetic prions revealed that a phenomenon very similar to strain adaptation was observed without changing host species [16], [17]. When rPrP fibrils gave rise to prion diseases, the disease phenotype continued to evolve for as long as four serial passages [16], [17]. Adaptation consisted of a long clinically silent stage and was accompanied by transformation of PrP^Sc^ physical properties and neuropathological features [14], [16]. In the absence of even miniscule amounts of PrP^Sc^ in rPrP fibril preparations, such a long adaptation period was attributed to structural transformation of self-replicating states and evolution of authentic PrP^Sc^ via deformed templating [4]. These studies raised the possibility that a deformed templating mechanism might also be involved in prion strain adaptation that accompanies cross-species transmission.

## Strain Mutation versus Norm of Reaction

Deformed templating is one of the mechanisms that account for prion strain mutation and evolution. Defining strain mutation could be difficult when prions are replicated *in vitro*, because PrP^Sc^ often undergoes gradual transformation in cultured cells or under different PMCA formats [2], [3], [18]–[20]. For instance, adding the glycosylation inhibitor, swainsonin, caused gradual transformation of prion strains in cultured cells [2], [20]. Dramatic transformations of PrP^Sc^ proteinase resistance profiles were observed in PMCA with partially deglycosylated substrates [18]. Such transformations are often reversible and do not lead to stable changes in disease phenotype when tested in animals [18]–[20]. While PrP^Sc^ transformations in diverse cellular or biochemical environments are indicative of its dynamic nature, they should not be confused with actual strain mutation.

With the finding of PrP^Sc^ plasticity due to replication in diverse cellular or PMCA environments, it is worthwhile to compare this phenomenon to a norm of reaction, a concept that describes phenotypic variations of a single genotype across a range of environments. The concepts of norm of reaction and phenotypic plasticity were introduced into population genetics to describe variations in phenotype and the ability of an organism to change its phenotype, respectively, in response to changes in the environment. For instance, plants can acquire multiple morphologically distinct phenotypes within a single genotype to fit into a diverse range of environments. Noteworthily, phenotypic plasticity is not attributed to mutations but to an intrinsic norm of reaction. A concept analogous to norm of reaction could be useful for describing variations in PrP^Sc^ features observed across diverse replication environments, such as different cultured cells and PMCA formats that do not lead to stable changes in disease phenotype. In other words, norm of reaction is defined as variation of those strain features across diverse replication environments that are not essential for defining strain-ness.
